# Effect of Exercise-Induced Enhancement of the Leg-Extensor Muscle-Tendon Unit Capacities on Ambulatory Mechanics and Knee Osteoarthritis Markers in the Elderly

**DOI:** 10.1371/journal.pone.0099330

**Published:** 2014-06-06

**Authors:** Kiros Karamanidis, Kai Daniel Oberländer, Anja Niehoff, Gaspar Epro, Gert-Peter Brüggemann

**Affiliations:** 1 Institute of Biomechanics and Orthopaedics, German Sport University of Cologne, Cologne, Germany; 2 Department of Mathematics and Technology, University of Applied Sciences Koblenz, RheinAhrCampus Remagen, Koblenz, Germany; 3 Institute of Movement and Sport Gerontology, German Sport University Cologne, Cologne, Germany; 4 Cologne Center for Musculoskeletal Biomechanics, Medical Faculty, University of Cologne, Cologne, Germany; University of Zurich, Switzerland

## Abstract

**Objective:**

Leg-extensor muscle weakness could be a key component in knee joint degeneration in the elderly because it may result in altered muscular control during locomotion influencing the mechanical environment within the joint. This work aimed to examine whether an exercise-induced enhancement of the triceps surae (TS) and quadriceps femoris (QF) muscle-tendon unit (MTU) capacities would affect mechanical and biological markers for knee osteoarthritis in the elderly.

**Methods:**

Twelve older women completed a 14-week TS and QF MTU exercise intervention, which had already been established as increasing muscle strength and tendon stiffness. Locomotion mechanics and serum cartilage oligomeric matrix protein (COMP) levels were examined during incline walking. MTU mechanical properties were assessed using simultaneously ultrasonography and dynamometry.

**Results:**

Post exercise intervention, the elderly had higher TS and QF contractile strength and tendon-aponeurosis stiffness. Regarding the incline gait task, the subjects demonstrated a lower external knee adduction moment and lower knee adduction angular impulse during the stance phase post-intervention. Furthermore, post-intervention compared to pre-intervention, the elderly showed lower external hip adduction moment, but revealed higher plantarflexion pushoff moment. The changes in the external knee adduction moment were significantly correlated with the improvement in ankle pushoff function. Serum COMP concentration increased in response to the 0.5-h incline walking exercise with no differences in the magnitude of increment between pre- and post-intervention.

**Conclusions:**

This work emphasizes the important role played by the ankle pushoff function in knee joint mechanical loading during locomotion, and may justify the inclusion of the TS MTU in prevention programs aiming to positively influence specific mechanical markers for knee osteoarthritis in the elderly. However, the study was unable to show that COMP is amenable to change in the elderly following a 14-week exercise intervention and, therefore, the physiological benefit of improved muscle function for knee cartilage requires further investigation.

## Introduction

Tibiofemoral joint osteoarthritis (OA) is one of the most common musculoskeletal disorders among people over the age of 60 [1]. The disease most frequently affects the medial compartment of the joint, which leads to a progressive loss of articular cartilage, causing a devastating effect both on mobility and on independent living in the older population [1]. Linked to higher life expectancy and increasing demands among the elderly to remain active, effective intervention strategies for medial compartment knee OA are in demand and should be one of the major aims of health care.

There is increasing evidence that mechanical factors related to repetitive joint loading during locomotion may play a key role in the initiation and progression of knee OA in the elderly [2], [3]. In particular, an altered mechanical environment within the joint and an increased proportion of the compressive loading of the tibiofemoral joint on the medial compartment have been suggested as contributing factors for cartilage breakdown in the elderly [4], [5], [6]. A valid proxy for the mechanical load distribution at the tibiofemoral joint during *in vivo* motion is the external knee adduction moment, i.e. a higher external knee adduction moment indicates higher mechanical loads in the medial compared to the lateral compartment [2], [7]. The presence, rate and severity of medial compartment knee OA in the elderly has been associated with the magnitude of knee adduction moments during the activities of daily living [4], [5], [6]. Of particular interest to the pathogenesis, prevention and therapy of medial compartment knee OA in the elderly, therefore, is the ability to counteract excessive external knee adduction moments during the activities of daily living.

Next to the point of force application, the magnitude of the knee adduction moment during locomotion is determined by the magnitudes of the vertical and mediolateral components of the ground reaction force (GRF) [2]. This is because these two components define the orientation of the GRF in the frontal plane and, hence, the distance of the GRF vector to the knee joint centre [2]. The individual muscles of the lower-limb control the GRF vector during locomotion and the lower-limb muscles are, therefore, considered to be the main contributors to the external knee adduction moment [8], [9], [10], [11]. The skeleton of the lower extremity is a system of jointed segments which are dynamically coupled. That is to say, each muscle force is transmitted by all the joints, and thus, each muscle accelerates all the body segments simultaneously during the ground contact phase [9], [11]. Recent modelling studies [9], [11] have reported that the external adduction moment at the knee arises mainly from the actions of three major muscle groups: the triceps surae (TS), the quadriceps femoris (QF) and the hip abductors such as the gluteus medius. While the hip abductors have been found to increase the mediolateral component of the GRF (i.e. more medially directed GRF vector) and hence the external knee adduction moment while walking and running, the TS and the QF act to provide a relatively large resistance to the external knee adduction moment (i.e. more laterally directed GRF vector) [9], [11]. This is an important observation because, beyond the fifth decade of human life, TS and QF muscle-tendon unit (MTU) capacities gradually decline (i.e. lower muscle strength, lower tendon stiffness) [12]. Thus, TS and QF MTU weakness could play a role in the pathogenesis of knee OA in the elderly by altering the load distribution at the knee joint during locomotion.

Investigating this issue, Karamanidis & Arampatzis [13] recently reported that, compared to younger adults, older adults use their ankle plantarflexors and knee extensors less and their hip abductors more while ascending stairs and ramps, aiming to compensate for the decline in their TS and QF MTU capacities. The consequence of this readjustment of muscular output in the lower extremity was that the older adults directed the GRF vector more medially in relation to the knee joint centre, thereby increasing the magnitude of the external adduction moment at the knee [13]. The readjustment of the muscular output and as a consequence the load redistribution at the knee joint (mechanical changes) in the elderly seems to be initiated by the TS and QF MTU weakness (biological changes). Thus, increasing the capacities of the TS and QF MTUs may be a promising approach to reducing mechanical risk factors for knee OA caused by excessive loads in the elderly. To our knowledge, there has been no study testing whether an experimental enhancement of the TS and QF MTU capacities (i.e. increased muscle strength, increased tendon stiffness) would lead to a decrease in the magnitude of the external knee adduction moment in the elderly during locomotion.

In the present study we analyzed incline walking because this motor task is frequently performed during daily activities and puts high demands on the musculoskeletal system. The aim of this work was, therefore, to test whether frontal-plane knee and hip joint kinetics during incline walking can be modified by experimentally increasing the capacities of the TS and QF MTUs in older adults. Physical exercise with a high magnitude of mechanical stress and strain imposed on the MTUs is known to trigger adaptive mechanical changes in skeletal muscles and collagenous tissues [14] and is regarded, therefore, as an effective method of experimentally increasing muscle strength and tendon stiffness in older adults [15]. On the basis of the prediction of the walking simulation studies [9], [11], we hypothesised that older adults would benefit from such an exercise-induced enhancement of the TS and QF MTU capacities, reflected in increased ankle plantarflexor and knee extensor joint moment outputs during incline walking, and redirection of the GRF vector in the frontal plane, thereby reducing the magnitude of external knee adduction moment. In order to determine potential changes in cartilage response in the elderly due to the motor task reorganization, serum levels of cartilage oligomeric matrix protein (COMP) as a biomarker of cartilage metabolism were analyzed after a controlled incline walking exercise on the treadmill. COMP is currently being studied as a serum marker for cartilage degeneration in OA and knee joint disease [16], [17], and its serum level increases with cyclic mechanical loading [18], [19], [20]. Therefore, we additionally aimed to test the hypothesis that the consequences of the lower ambulatory knee joint loading in the elderly after the improvement of the TS and QF MTU capacities would influence cartilage metabolism by decreasing the serum COMP concentration after the cyclic loading exercise.

## Methods

### Participants and experiment design

The experimental study was conducted on 12 older female subjects aged between 64 and 75 years (mean ± SD: 69±4 years; body mass: 68±7 Kg; body height: 160±5 cm). All subjects were screened to exclude any symptoms or signs of neuromuscular or skeletal impairments including knee joint pain and any radiographic knee OA symptoms documented by a specialist orthopaedic practitioner. Healthy subjects were included in this study because OA patients can functionally adapt to pathological joint changes [21]. Approval was obtained from the German Sport University's Committee for the Protection of Human Subjects according to the Helsinki Declaration. Further, the participants provided their written informed consent to participate in this study.

In order to increase TS and QF muscle strength and tendon stiffness we applied an exercise intervention which had already been established as increasing muscle strength and tendon stiffness in the elderly [15], consisting of a progressive 14-week MTU resistance exercise. Before (pre) and after (post) completing the MTU exercise intervention, each subject was tested over three days with respect to the following main outcome measures: (i) incline walking mechanics, (ii) changes in serum COMP concentration after a 0.5-h incline walking exercise, and (iii) TS and QF muscle strength and tendon stiffness.

### Analysis of incline walking mechanics

All subjects ascended a purpose-built three-step ramp (gradient: ∼21%) in a step-over manner as described earlier [13]. The participants were requested to walk at their self-selected preferred speed and slightly faster than preferred speed. Per participant and speed, at least five valid trials were collected. Ground reaction forces were determined using three force plates (Kistler, Winterthur, CH, 60×40 cm, 1080 Hz) embedded in the ground – two for the right leg and one for the left leg [13]. Kinematics were obtained using a Vicon Nexus motion capture system (Vicon Motion Systems, Oxford, United Kingdom) with 14 infrared cameras operating at a sampling rate of 120 Hz. The second step on the ramp was considered for further analysis, i.e. left leg [13].

To track complete body kinematics during incline walking, a multisegment system of 39 reflective markers (diameter 14 mm) was used and classified into a soft-tissue deformation optimised full-body model of 13 segments (feet, shanks, thighs, forearms, upper arms, pelvis, torso and head) [22]. To reduce soft tissue artefacts during locomotion, the marker positions of the lower extremity and of the pelvis were optimised with respect to the neutral position by a singular value decomposition algorithm provided by Söderkvist & Wedin [23]. The resultant (external) joint moments at the ankle, knee and hip of the left leg were calculated through inverse dynamics [24]. The masses and moments of inertia of the various body segments were calculated using the data provided by Zatsiorsky & Selujanov [25].

### Analysis of serum COMP concentration

For the serum COMP analyses, all subjects had to walk an incline ramp condition (gradient: ∼14%) on a motor-driven treadmill with a belt speed of 0.7 m•s^−1^ (pulsar 4.0, h/p/cosmos, Nussdorf-Traunstein, Germany) for 0.5-h. Familiarization with the treadmill was carried out for each subject three to four days before measurement. Subjects were asked to limit their physical activity for 24-h prior to the experiment and all measurements took place within 1.5-h of waking. Subjects were in a relaxed seated position for 0.5-h before the first blood sample was taken, prior to the gait exercise. Blood was collected in serum separation tubes by venous puncture performed by medical personal immediately before (directly after the 0.5-h seated; baseline), immediately after, and 0.5- and 1.0-h after the 0.5-h incline walking exercise. Immediately after the 0.5-h walking exercise all subjects lay prone on a daybed and maintained a relaxed body position for 1.0-h. Serum levels of COMP concentrations were determined using a commercial enzyme-linked immunosorbent assay (ELISA; COMP Elisa, ANAMAR Medical AG, Lund, Sweden) as described earlier [20]. A four-parameter regression curve calculated with Sigma Plot 8.0 (Systat software Inc., San Jose, USA) was used to define the standard curve of light absorbance and COMP concentrations, and to interpolate unknown serum COMP concentrations of the samples. The samples were analyzed in duplicate. Differences due to inter-assay variation were excluded by testing all samples of each participant on the same plate. The intra-assay and inter-assay coefficients of variation were 1.9% and 2.7%, and the detection limit was <0.1 U/l [26].

### Analysis of TS and QF muscle strength and tendon stiffness

To examine the MTU mechanical properties, all subjects performed isometric maximal voluntary ankle plantarflexion and knee extension contractions on a dynamometer (Biodex Medical Systems. Inc., Shirley, New York, USA). Axis misalignment between dynamometer and ankle or knee joint during contraction, gravitational moments and the moments arising from antagonistic muscles during the ankle plantarflexion and knee extension efforts were taken into consideration using 11 Vicon cameras operating at 120 Hz (MX-F40 NIR16, Vicon Motion Systems, Oxford, United Kingdom) and surface electromyography (EMG; Biovision, Wehrheim, Germany) device [27], [28], [29]. The antagonistic moment of the tibialis anterior or the hamstring muscles during contractions was estimated by establishing a relationship between EMG amplitude and exerted moment for the tibialis anterior or the hamstring whilst working as an agonist [29].

For the analysis of tendon mechanical properties, the elongation of the gastrocnemius medialis and vastus lateralis tendon and aponeurosis during contraction was visually reproduced using a 7.5 MHz linear array ultrasound probe (Aloka, Tokyo, Japan; ultrasound system: SSD-4000). The effect of inevitable joint angular rotation on the elongation of the tendon and aponeurosis during the loading phase was taken into account by capturing the motion of the tendons and aponeuroses from the gastrocnemius medialis and vastus lateralis during a passive (inactive condition) motion of the ankle or the knee joint [30]. The strain values of the gastrocnemius medialis and vastus lateralis tendon and aponeurosis during isometric maximal voluntary contraction were calculated at every 200 N and at maximal calculated tendon force respectively. The tendon force was calculated by dividing the ankle or knee joint moment by the corresponding tendon moment arm [31], [32]. The normalized stiffness of the TS and QF tendons and aponeurosis was calculated as the ratio of the increase in the calculated tendon force and the increase in the tendon-aponeurosis strain from 50 to 100% of the maximum tendon force. Tendon-aponeurosis resting length was determined at rest (relaxed muscles) and identified on the ultrasound images using specific joint angle configurations as described elsewhere [33], [34] (for the gastrocnemius medialis MTU the ankle joint was plantarflexed at 20° and the knee was in full extension; for the vastus lateralis MTU the knee and hip joint was flexed at 50° and 40°, respectively). The specific joint angles were chosen in order to reduce passive joint moments almost to zero [35] and, hence, to allow slackness of the corresponding tendon and aponeurosis. De Monte and colleagues [36] recently reported the existence of slackness in the inactive gastrocnemius medialis MTU when using these specific positions. We used the relationship between calculated tendon force and tendon-aponeurosis strain because different rest lengths may cause difficulties for the comparison between pre and post measurements. This is because different rest lengths influence the measured elongation of the tendon and aponeurosis and thus affect the estimate of the stiffness (the relationship between tendon force and elongation) of the tendon [33]. Therefore, we calculated the tendon-aponeurosis stiffness by using the relationship between calculated tendon force and strain and called this normalized tendon stiffness [34].

### TS and QF MTU exercise intervention

Participants completed a TS and QF MTU resistance exercise intervention under the supervision of several study investigators two (week one to week seven) or three (week eight to week 14) times per week for 14 weeks (approximately 1.5-h per session). Repetition of all exercises was progressed to a maximum of 30 (3 sets, 10 repetitions each) as tolerated by the subjects in order to use high magnitudes of mechanical stress and strain imposed on the MTUs and, hence, to increase muscle strength and tendon stiffness [14], [15]. Weights or difficulty level of the exercise were added or increased when subjects could complete 30 repetitions. The following exercises were used: ankle plantarflexion contractions at different TS MTU lengths (i.e. while sitting and while standing) on weight machines and on wall bars (with and without additional weights or resistance applied to the back), hopping in place (with and without additional weights or resistance applied to the back), seated leg press and knee extension contractions on weight machines, and squatting position exercises.

### Statistical analysis

To determine potential differences in TS and QF MTU mechanical properties between pre- and post-intervention, we used a one-way analysis of variance (ANOVA) with intervention (pre and post) as a factor. In order to get relevant information about locomotion mechanics in the initial, mid and final parts of the stance phases, joint kinetic variables were determined at ten intervals over the entire stance phase. A two-way ANOVA with repeated measures, with intervention (pre and post) and time window (examined intervals over the stance phase) as factors, was used in order to check for pre- and post-intervention differences of lower extremity joint mechanics during incline walking. For the spatio-temporal gait parameters and the external knee adduction angular impulse during stance phase, we used a one-way ANOVA with intervention as a factor. Before applying our ANOVA model, we tested the normal distribution of the examined variables using the Kolmogorov–Smirnov Test, which revealed that our data were normally distributed (P values were >0.05). If a significant interaction effect (intervention x time window) in lower extremity joint mechanics was found, we applied simple contrasts to further investigate whether the outcome measures at a certain time window differed between pre- and post-intervention. For each subject, the three trials with the most closely matched pre- and post-intervention speed were considered (identified by gait cadence), and the mean values from these trials were used for the statistics. Potential differences in serum COMP concentration were checked by using a two-factor ANOVA with repeated measurements, with intervention (pre and post) and time (immediately before [−0.5-h, baseline], immediately after [0-h], and 0.5- and 1.0-h after the 0.5-h incline walking exercise) as factors. Duncan's post-hoc comparison was performed when a significant main effect was detected. The level of significance was set for P-values less than 0.05. Statistically significant results in incline walking mechanics and serum COMP concentration were analyzed for the effect size, using Cohen's *d* [37]. Relationships between variables have been examined using a linear regression model. All results in the text, tables and figures are presented as mean and standard error of mean (mean and SEM).

## Results

### Changes in TS and QF muscle strength and tendon stiffness

**Figure 1:**
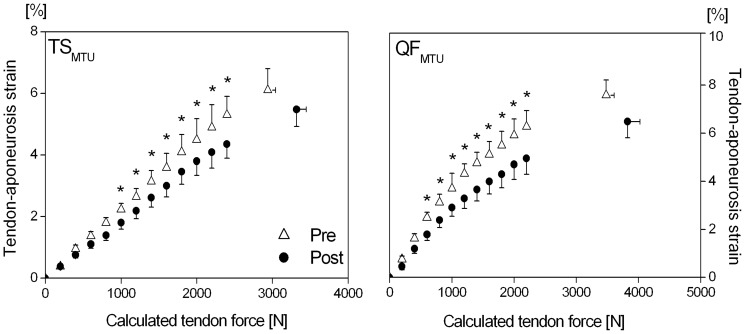
Tendon-aponeurosis strain of the gastrocnemius medialis (TS_MTU_) and vastus lateralis (QF_MTU_) at every 200 N of the calculated Achilles and patella tendon force during isometric maximal voluntary ankle plantarflexion and knee extension contractions determined before (pre) and after (post) the triceps surae and quadriceps femoris muscle-tendon unit exercise-intervention (means and SE). ^*^: Statistically significant differences between pre and post (P<0.05).

With regard to the contractile strength, the examined subjects showed significantly (P<0.05) higher maximal isometric ankle plantarflexion (pre: 2.01±0.13 Nm kg^−1^ *vs.* post: 2.28±0.12 Nm kg^−1^) and knee extension joint moments (2.28±0.08 Nm kg^−1^ *vs.* 2.49±0.09 Nm kg^−1^) following the intervention. For a given tendon force (every 200 N), the strain values of the TS and QF tendon and aponeurosis were significantly (P<0.05) lower post- compared to pre-intervention (Fig. 1). Thus, as for the gains in the contractile strength, post-intervention normalized tendon stiffness was significantly higher (P<0.05) for both MTUs compared to pre-intervention (TS: 51.4±3.8 kN strain^−1^ *vs.* 62.4±3.6 kN strain^−1^; QF: 76.5±5.1 kN strain^−1^ *vs.* 89.9±4.8 kN strain^−1^).

### Changes in incline walking mechanics

**Figure 2:**
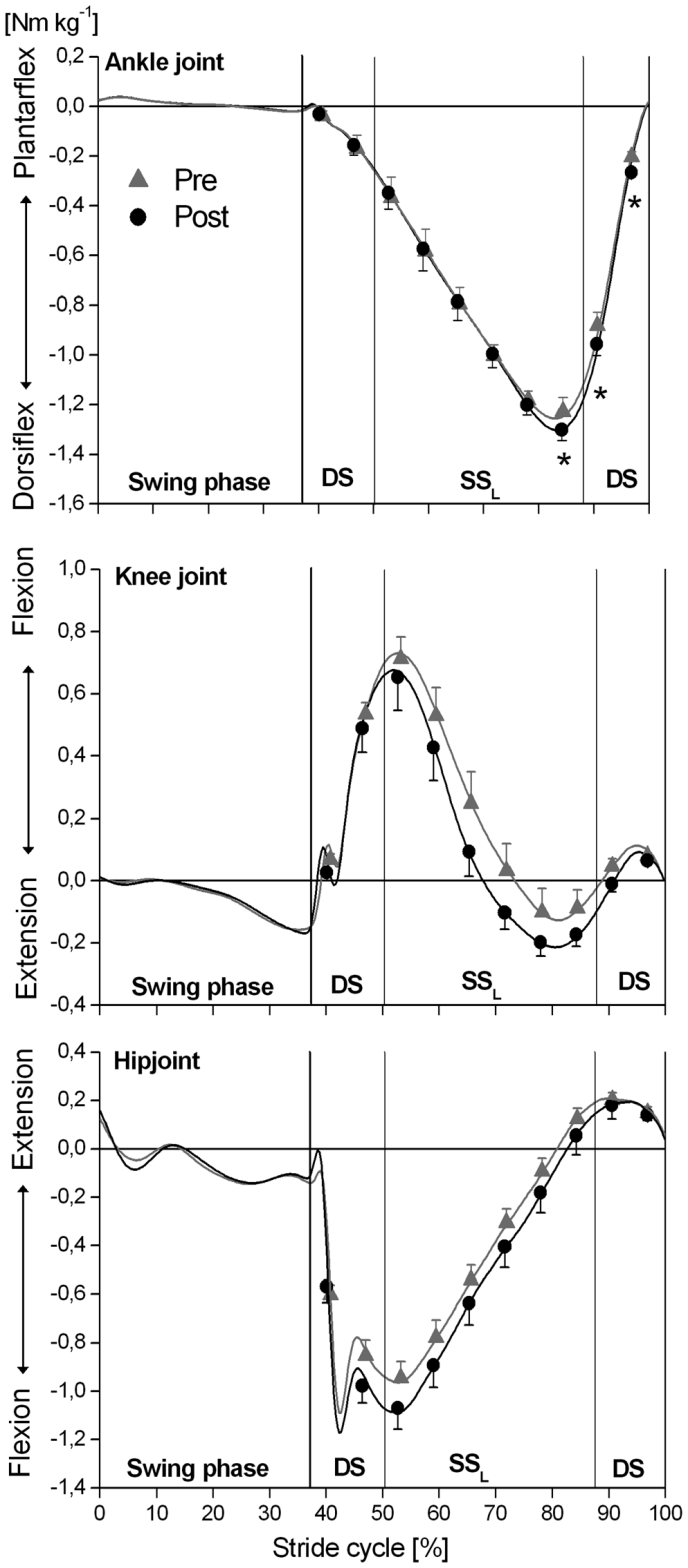
External joint moments at the ankle, knee and hip in the sagittal plane at different phases of the ground contact phase while incline walking determined before (pre) and after (post) the triceps surae and quadriceps femoris muscle-tendon unit exercise-intervention (means and SE). The x-axis was normalized from 0 to 100% of a stride cycle. The vertical solid lines represent the instants of touchdown (TD) and take off (TO) of the left and right foot. DS: double support phase; SS_L_: single support phase of the analyzed left leg. ^*^: Statistically significant differences between pre and post (P<0.05).

**Figure 3:**
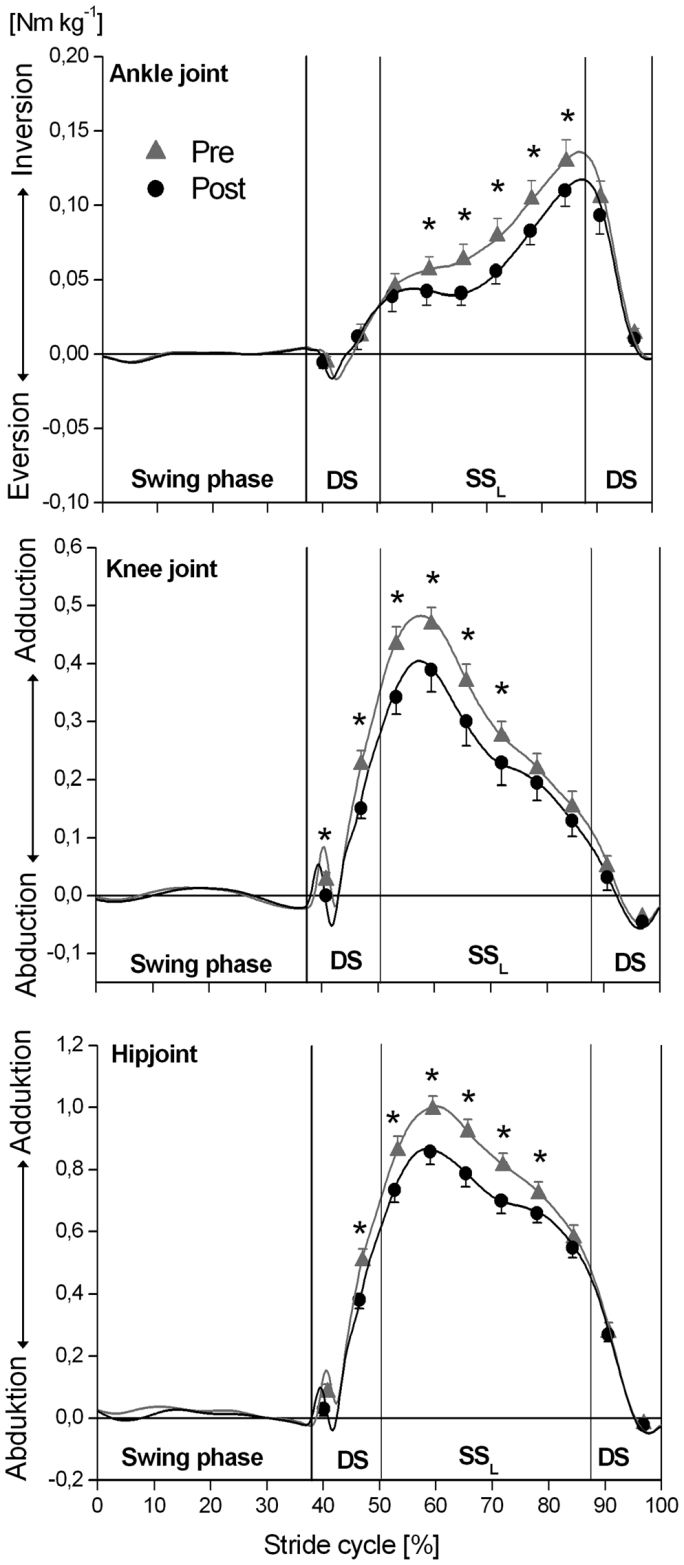
External joint moments at the ankle, knee and hip in the frontal plane at different phases of the ground contact phase while incline walking determined before (pre) and after (post) the triceps surae and quadriceps femoris muscle-tendon unit exercise-intervention (means and SE). The x-axis was normalized from 0 to 100% of a stride cycle. The vertical solid lines represent the instants of touchdown (TD) and take off (TO) of the left and right foot. DS: double support phase; SS_L_: single support phase of the analyzed left leg. ^*^: Statistically significant differences between pre and post (P<0.05).

**Figure 4:**
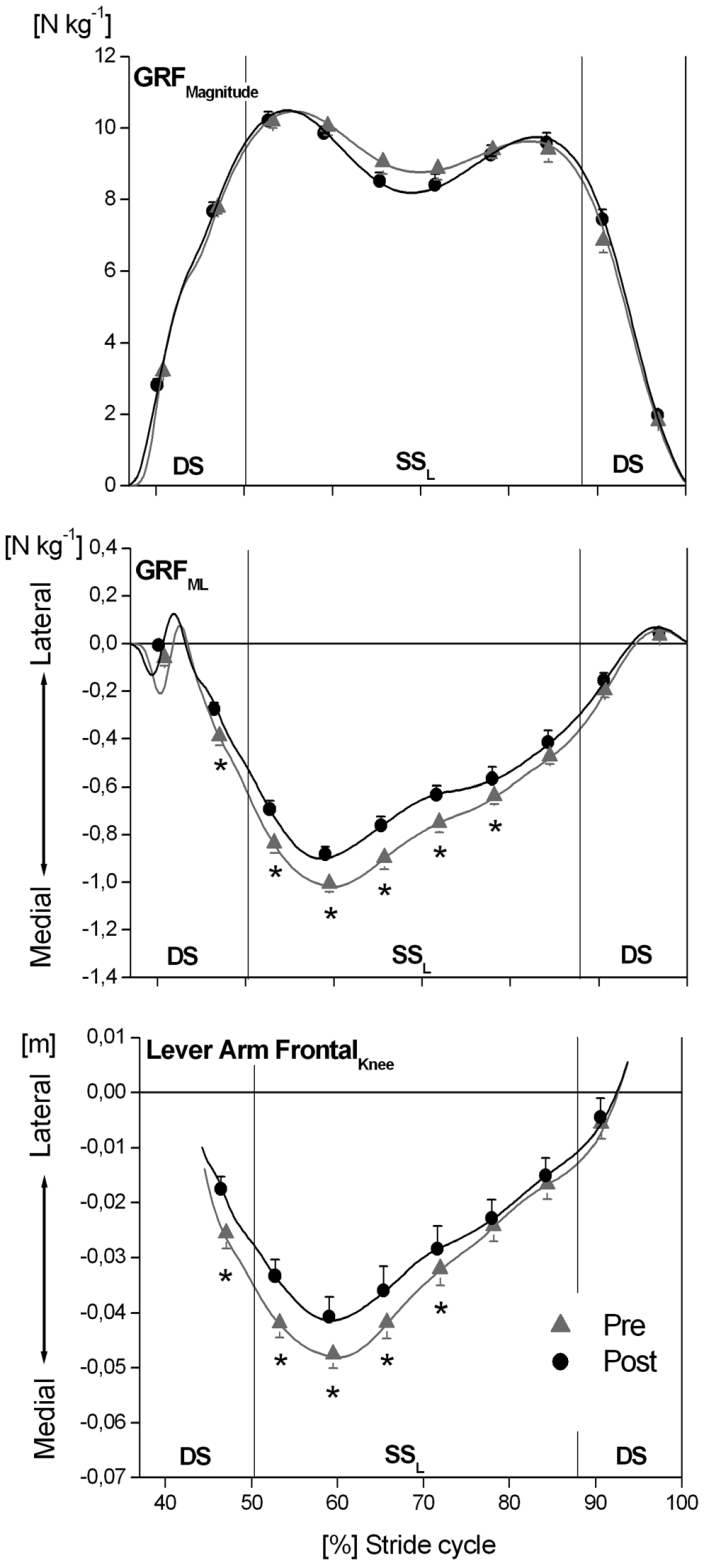
Magnitude of the ground reaction force vector (GRF_Magnitude_), mediolateral component of the GRF (GRF_ML_) and lever arm of the GRF acting about the knee joint at the frontal plane (Lever Arm Frontal_Knee_) while incline walking determined before (pre) and after (post) the triceps surae and quadriceps femoris muscle-tendon unit exercise-intervention (means and SE). The x-axis was normalized from 0 to 100% of a stride cycle. The vertical solid lines represent the instants of touchdown (TD) and take off (TO) of the left and right foot. DS: double support phase; SS_L_: single support phase of the analyzed left leg. Lateral-medial Lever Arm Frontal_Knee_ refers to the position of the line of action of the GRF relative to the knee joint centre. Lever Arm Frontal_Knee_ was not determined for the first and last 10% of the support phase because of the low GRFs and the consequently unreliable calculation of Lever Arm Frontal_Knee_. ^*^: Statistically significant differences between pre and post (P<0.05).

A significant (P<0.05) intervention x time window effect was found for the external dorsiflexion-plantar flexion ankle joint moment (Fig. 2), and for all examined kinetic outcome measures in the frontal plane (moments at the ankle, knee and hip joint, lever arm of the GRF acting about the knee joint and mediolateral component of the GRF; Fig. 3 and Fig. 4). The simple contrast test showed that at post- in comparison to pre-intervention, the older adults had significantly (P<0.05) higher external ankle dorsiflexion moments in the terminal portion of the stance phase (effect size >0.4) but revealed no significant (P>0.05) intervention effects on sagittal plane ankle joint moments in the initial and mid portions of the stance phase (Fig. 2; Table S1 in File S1). Concerning the joint moments in the frontal plane, the older adults showed significantly (P<0.05) lower external ankle inversion moments (effect size >0.5) and lower external adduction moments at the knee and hip joints (effect size >0.7) in the initial and mid portions of the stance phase post-intervention (Fig. 3; Table S1–S3 in File S1). Furthermore, the external knee adduction angular impulse (calculated as the integral of the external knee adduction moment over the time of the stance phase) showed significantly (P<0.05) lower values following the MTU intervention (pre: 123.2±10.8 Nm kg^−1^ ms vs. post: 99.8±11.9 Nm kg^−1^ ms) with a pre/post effect size of 0.7.

Post intervention, the mediolateral component of the GRF (Fig. 4; Table S4 in File S1) as well as the lever arms of the GRF acting about the knee joint in the frontal plane (Fig. 4; Table S5 in File S1) were significantly (P<0.05) lower in the initial and mid portions of the stance phase compared to pre-intervention. For both parameters the pre/post effect size was above 0.6. There was no significant (P>0.05) intervention effect or intervention x time window effect on the flexion-extension moments at the knee and hip joints during the stance phase (Fig. 2; Table S2–S3 in File S1) or on the magnitude of the GRF vector during incline walking (Fig. 4; Table S4 in File S1).

**Figure 5:**
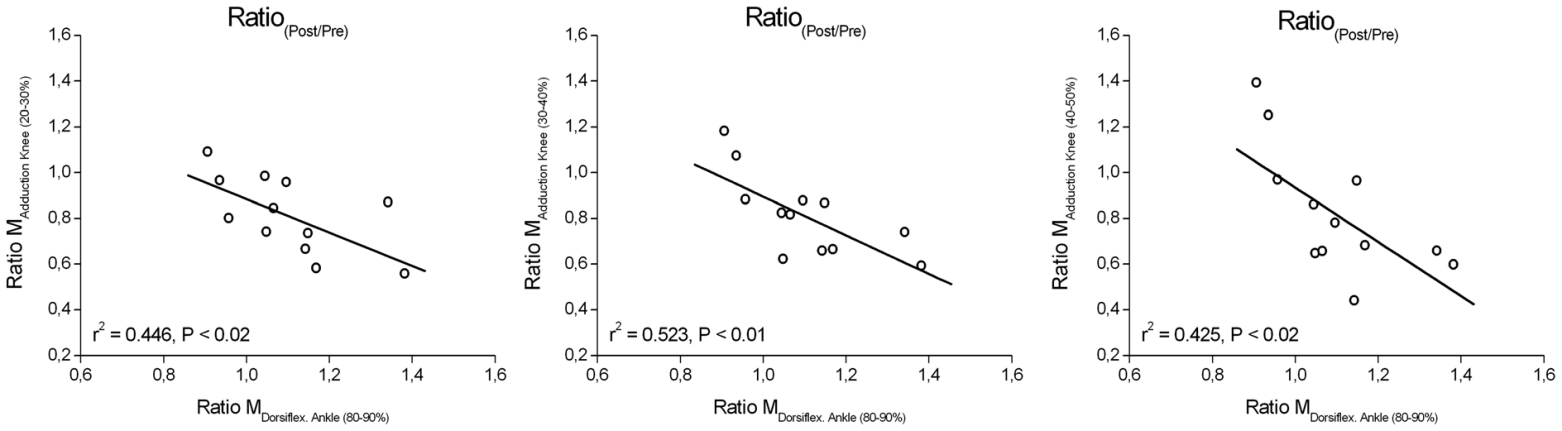
Relationship between the ratio post- to pre-intervention in external knee adduction moments (Ratio M_Adduction_ _Knee_) in the initial and mid portions of the stance phase (between 20% and 50% of the stance phase; leading leg) and the ratio post- to pre-intervention in external ankle dorsiflexion moment (Ratio M_Dorsiflex_. _Ankle_) in the terminal portion of the stance phase (between 80% and 90% of the stance phase; trailing leg) while incline walking.

We found a significant (P<0.05) correlation between the ratio post- to pre-intervention in external knee adduction moments in the initial and mid portions of the stance phase and the ratio post- to pre-intervention in external ankle dorsiflexion moment in the terminal portion of the stance phase (correlation coefficient from −0.65 to −0.72, P<0.05; Fig. 5). Furthermore, the ratio post- to pre-intervention in external knee adduction moment in the initial and mid portions of the stance phase showed a significant (P<0.05) correlation with the ratio post- to pre-intervention in lever arm of the GRF acting about the knee (0.80<r<0.99) and with the ratio post- to pre-intervention in hip joint moment in the frontal plane (0.63<r<0.81). There was no statistically significant difference (P>0.05) between pre- and post-intervention in ground contact duration (544±13 ms *vs.* 552±16 ms) or swing duration (329±9 ms *vs.* 324±8 ms) and, hence, cadence (129.1±3.2 steps min^−1^ *vs.* 123.9±3.7 steps min^−1^).

### Changes in serum COMP concentration

The mean baseline serum COMP concentration was 12.1±1.2 U l^−1^and 11.7±0.8 U l^−1^ on the experimental day of pre and post MTU exercise intervention, respectively (Table S6 in File S1). These values could be classified in the category of increasing risk of aggressive joint destruction (>12 U l^−1^; AnaMar Medical AB) and may confirm an elevated risk for the development of OA and joint disease in the examined group of older women.

**Figure 6:**
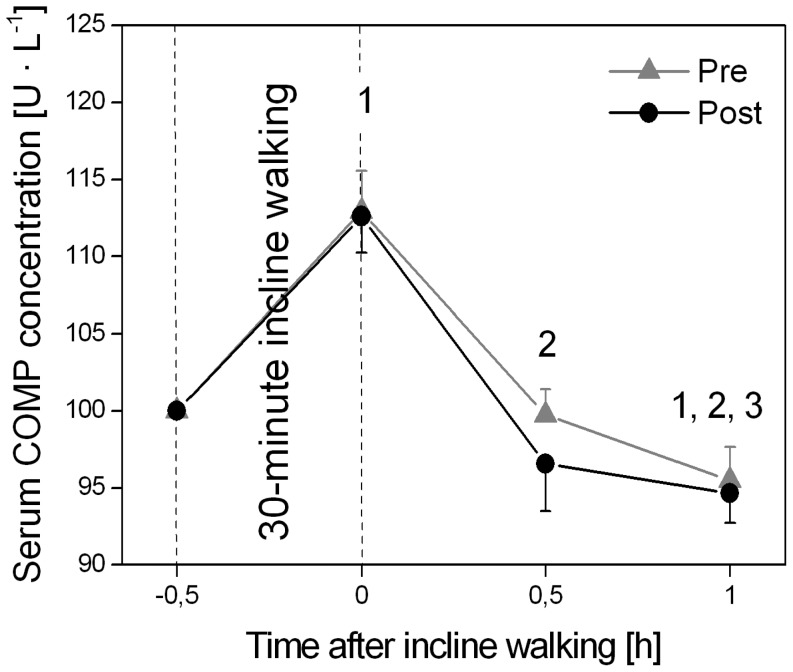
Serum Cartilage Oligomeric Matrix Protein (COMP) concentration before (−0.5-h; baseline), immediately after (0-h), and 0.5- and 1.0-h after the 0.5-h incline walking exercise determined before (pre) and after (post) the triceps surae and quadriceps femoris muscle-tendon unit exercise-intervention. Please note that serum COMP concentrations are presented as percent of baseline serum concentration and all statistical tests were performed on absolute values. There was no significant muscle-tendon unit exercise-intervention effect on serum COMP concentration (P = 0.23). ^1^: Statistically significant differences to −0.5-h (P<0.05). ^2^: Statistically significant differences to 0-h (P<0.05). ^3^. Statistically significant differences to 0.5-h (P<0.05).

Incline walking for 0.5-h had a significant (P<0.05) effect on serum COMP concentration (Fig. 6). The cyclic loading exercise leads to a significant (P<0.05) increase in serum COMP concentration with an average increase of 12.9% (pre MTU intervention) and 12.6% (post MTU intervention) with a pre/post effect size higher than 0.4. This increase of the serum COMP concentration immediately after walking exercise was not significantly (P>0.05) different between pre and post TS and QF MTU intervention (Fig. 6). Within a period of 1.0-h following the gait exercise, the COMP value decreased continuously and returned to a significantly (P<0.05) lower value compared to the baseline (Fig. 6). There was no significant (P>0.05) MTU exercise intervention effect and no intervention x time effect (P>0.05) on serum COMP concentration (Fig. 6).

## Discussion

In a previous study [13] we showed that older adults compensate for the degeneration of their TS and QF MTUs by shifting the relative distribution of the joint moments to the more proximal hip joint during locomotion. The cost of this compensation is that the older adults redistribute the mechanical load within the load-bearing regions of the knee, increasing the risk of cartilage breakdown. These experimental findings agree with the prediction from walking simulation studies [9], [11] that impaired TS and QF muscle function during locomotion alters the mechanical environment within the tibiofemoral joint and can therefore be a key component in knee joint degeneration in the elderly. The aim of this work was to test whether an exercise-induced adaptive enhancement of the TS and QF MTU capacities would influence mechanical and biological markers for knee OA in the elderly.

Post in comparison to pre physical exercise intervention, the examined older adults showed higher maximal voluntary isometric ankle plantarflexion and knee extension joint moments and higher tendon-aponeurosis stiffness at the TS and QF MTU. The above findings are in accordance with the literature [15] and support the view that in old age, not only muscles but also tendons are highly responsive to training and that a physical exercise intervention with a high magnitude of mechanical stress and strain imposed on the MTU can increase contractile strength and tendon-aponeurosis stiffness in the elderly. With regard to the incline gait task, the older adults demonstrated post- in comparison to pre-intervention, a lower magnitude of the external knee adduction joint moment in the initial and mid parts of the support phase. Furthermore, the older adults examined showed an approximately 20% reduction in the external knee adduction angular impulse during the stance phase post-intervention. In the literature, the external adduction moment and angular impulse at the knee has been noted for its potential role in the initiation and progression of medial compartment knee OA in the elderly [4], [5], [6]. Amin *et al.* [4] and Miyazaki *et al.* [6], for example, showed that greater adduction moment at the knee during locomotion contributes to the development of future chronic knee pain and is a valid predictor of radiographic disease progression in the medial compartment in the elderly. The current significant difference in the external knee adduction moment and knee adduction impulse was further analyzed in terms of effect size, which was concluded to be practically relevant (Cohen's *d* from 0.7 to 1.0). The identified changes in ambulatory knee joint mechanics and MTU mechanical properties, therefore, suggest that the examined older adults showed post 14-week exercise intervention, enhanced leg-extensor MTU capacities and some positive changes on specific mechanical markers for medial compartment knee OA.

The magnitude of the GRF vector during the first part of the support phase was not significantly different between pre- and post-intervention. Moreover, the ratio post- to pre-intervention in external knee adduction moment showed a high correlation with the ratio post- to pre-intervention in lever arm of the GRF acting about the knee joint in the frontal plane (correlation coefficient up to 0.99). Therefore, the reduction of the external knee adduction moment in the elderly was associated with changes in the lever arms. Previous studies have reported that the orientation of the foot with respect to the direction of progression can affect the frontal plane lever arm at the knee joint, and hence knee adduction moment [21]. However, we did not find any significant (P>0.05) changes in foot progression angle (pre: 4.7±1.2° *vs.* post: 5.1±1.1°; mean values during the entire stance phase). Our findings rather suggest that the lower knee adduction moment resulted from the decreased moment output from the hip abductors following the intervention. We found a significant relationship between the ratio post- to pre-intervention in external knee adduction moment and the ratio post- to pre-intervention in external hip adduction joint moment (r up to 0.81). The hip abductors act to increase the mediolateral component of the GRF, in particular during the single support phase [9], and an experimentally impaired gluteus medius muscle function has been shown to reduce the external knee adduction moment while walking [38]. A reasonable explanation for our findings is, therefore, that by decreasing the hip abduction moment output during the initial and mid portions of the stance phase (Fig. 3), the older adults decreased the mediolateral component of the GRF (Fig. 4), positioning the line of action of the GRF closer to the knee joint (Fig. 4) and thereby decreasing the external knee adduction moment post-intervention (Fig. 3).

The changes in knee and hip joint kinetics in the frontal plane occurred in the first part of the support phase, where the analysed left leg is the leading leg. During this part of the support phase, the pre- and post-intervention ankle, knee and hip joint kinetics in the sagittal plane were similar. In the terminal part of the support phase, where the analyzed left leg is the trailing leg, the test subjects demonstrated a higher external ankle dorsiflexion joint moment post-intervention. These findings suggest that post- in comparison to pre-intervention, the older adults modified their gait strategy while incline walking and made greater use of the ankle plantarflexors of their trailing leg during pushoff. Lewis & Ferris [39] reported that there is a direct trade-off between the impulsive push from the ankle joint of the trailing leg and the need for the hip muscles acting on the leading leg to contribute to support and forward progression while walking. Although the hip abductors are generally not considered to be sagittal-plane muscles, modelling studies [8], [9] showed that the gluteus medius muscle also makes a substantial contribution to forward and vertical progression while walking, especially during the single support phase. We argue, therefore, that the mechanism behind the decrement of the external knee adduction joint moment at the leading leg post-intervention was the increased ankle pushoff joint moment of the trailing leg; by increasing their ankle pushoff moment, the older adults decreased the need for ipsilateral hip abduction joint moment output during incline walking.

In line with this suggestion, we found a significant relationship between the decrement (ratio post to pre) of the external knee adduction moment during the first part of the support phase (i.e. leading leg) and the increment (ratio post to pre) of the external ankle dorsiflexion moment in the terminal portion of the stance phase (i.e. trailing leg). At the initiation of the single support phase, where the knee adduction moment reached the highest magnitudes (between 30 and 40% of the stance phase, Fig. 3), this relationship was strongest with r = −0.72 (Fig. 5). This means that those elderly subjects who, post-intervention, were more able to increase the ankle pushoff moment thereby reduced the external knee adduction moment more. Recent *in vivo* ultrasound measurements [40] reported that the TS of the trailing leg shows a low force-length-velocity potential because gastrocnemius medialis fascicle length is short and shortens rapidly during pushoff when incline walking. This implies a high task demand for the degenerated TS MTU in the elderly. We argue, therefore, that the exercise-induced adaptive enhancement of contractile strength and tendon stiffness in the test subjects provided more favourable conditions for the TS to generate higher ankle pushoff moments. The stiffer TS tendon induced by the intervention may result in a reduced excursion of the series elastic element during stance. Because the gastrocnemius medialis predominately shortens during the stance phase during incline walking [40], a smaller excursion of the series elastic element will reduce fascicle shortening and thus shortening velocity, enabling the TS muscle to generate higher pushoff moments due to the force-velocity relationship. The findings, thus, highlight the important role of the ankle pushoff function on frontal-plane knee and hip joint kinetics during locomotion, and may justify the inclusion of the TS MTU in prevention programs in an effort to reduce some specific mechanical risk factors that contribute to knee joint disease in the older population.

Despite the identified altered ambulatory mechanics, we were unable to determine significant changes in serum COMP concentrations following the MTU intervention within the current analysed subject group. COMP is an established biomarker for joint degeneration and cartilage thinning [16], [17], and its serum level increases with mechanical loading [18], [19], [20]. Erhart-Hledik *et al.* [16], for example, showed that a short-term change in serum COMP concentration induced by a mechanical stimulus is associated with long-term degenerative morphological changes in cartilage at the tibiofemoral joint. The stimulus-response model used in the current study (i.e. 0.5-h incline walking at 0.7 m•s^−1^ with a gradient of 14%) demonstrates the ability of a relatively brief mechanical stimulus to produce a short-term biological response in biomarker concentration in the elderly population. However, comparing test results both pre and post the MTU exercise intervention, we were unable to detect significant differences in either the baseline changes in serum COMP levels, or in the duration of the increased serum COMP concentration in response to the 0.5-h incline walking exercise. Thus, the current data failed to verify the hypothesis that serum COMP might serve as a marker for mechanical outcome measures in the elderly following a 14-week MTU exercise intervention. Given the inherent variability in serum COMP concentration measures and that the blood serum markers are affected by a larger number of variables, we can only speculate that this result is likely to be a reflection of the small sample size and reduced ability to detect significant differences in serum COMP concentration. However, the results may also imply that the observed changes in the external knee adduction moment were not relevant for cartilage physiology within the examined subject group.

A further relevant drawback that needs to be considered when interpreting the current findings is that the homeostatic response of muscle, tendon and cartilage subjected to mechanical loading may be represented by different curves. It is well established that the adaptive response of different biological tissues to mechanical loading can follow different time patterns and that cartilage is less sensitive than skeletal muscle to a mechanical stimulus. Thus, in addition, we may speculate that the mechanical stimulus induced by the changes in ambulatory knee joint mechanics post in comparison to pre the 14-week physical exercise intervention was too low in amplitude and/or too short in duration to provoke measurable changes in cartilage metabolism with respect to serum COMP concentration in the examined group of older individuals. To our knowledge there is only limited data in the literature about the effect of long-term leg-extensor MTU therapeutic intervention programs on biomarker levels in the elderly. Therefore, additional studies are needed with perhaps longer intervention durations to determine the effect of improved TS and QF MTU function on serum COMP concentration and to examine the physiological relevance for the medial compartment cartilage of the biomechanical parameters studied in the elderly. However, although it was not possible to conclude from measuring the serum COMP concentration that the TS and QF MTU intervention improved joint structures, the finding of no increase in levels of serum COMP concentration over the course of the study indicates that the leg-extensor MTU exercise intervention, involving as it did high magnitudes of mechanical stress, did not result in measurable harm to the joints. This was a potential concern with older adults in an intervention program that included TS and QF MTU strengthening exercises involving periods of strong increase in joint mechanical loading.

**Figure 7:**
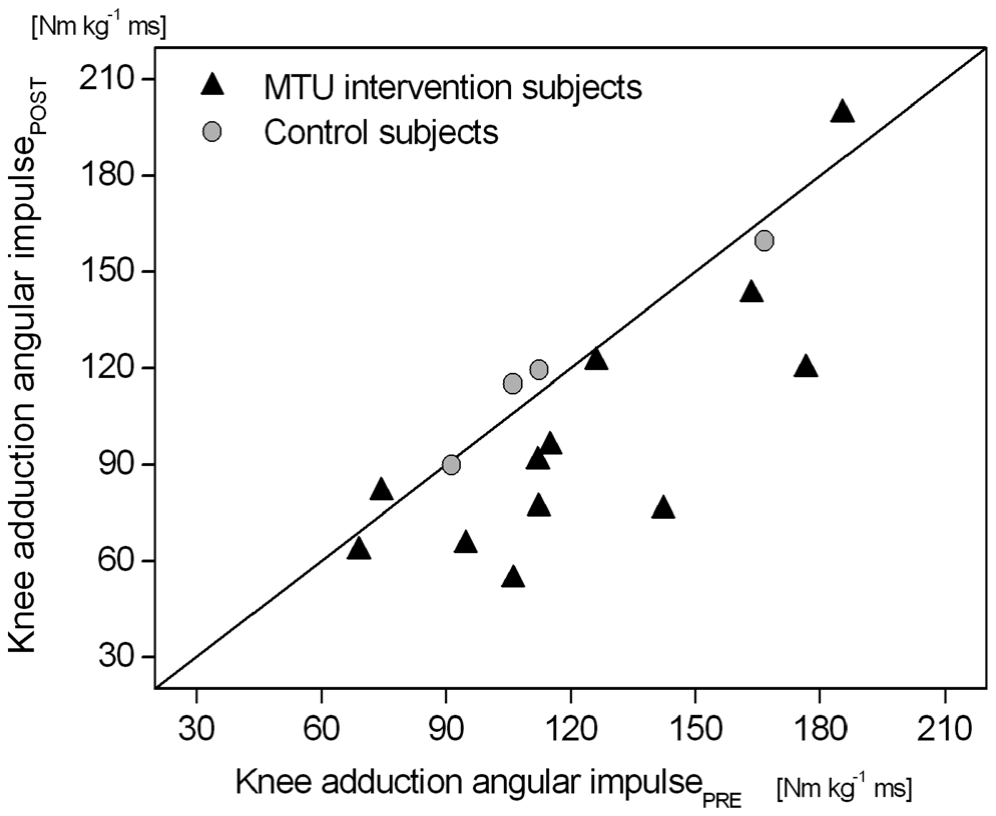
External knee adduction angular impulse during the stance phase while incline walking for the 12 intervention subjects and the 4 control subjects measured before (PRE) and after (POST) the 14-week exercise duration, with line of equality. The plot illustrates that the degree of agreement in the external knee adduction impulse between pre and post measurements was higher for the control group than for the intervention group.

A major limitation of this study is the lack of a control group. We originally started the empirical study with 26 older women (15 subjects in the intervention group and 11 subjects in the control group). Unfortunately, for a variety of reasons, a relatively large number of women in the control group dropped out during the 14-week period. Only four older women successfully completed all measurements and we therefore decided not to include the control group in our statistics. However, when considering the individual data of these four older women, we can suggest that the degree of agreement in gait characteristics between pre and post measurements was reasonably high. Exemplarily, in figure 7 we plotted the external knee adduction impulse using the individual data of the four control and the 12 intervention subjects and drew the line of equality on which all points would lie if the two meters gave exactly the same reading every time. The plot shows that the degree of agreement in the external knee adduction impulse between pre and post measurements was clearly higher for the control group than for the intervention group (Fig. 7). Moreover, for the external knee adduction moment the effect size within the intervention group was up to 1 of the standard deviation indicating that the magnitude of the difference between pre and post measurement was large. Additional studies are needed to examine the effectiveness of an exercise-induced enhancement in TS and QF contractile strength and tendon-aponeurosis stiffness in the elderly and to confirm that such gait improvements can be attributed predominantly to the training regime.

In conclusion, the older adults examined in the current study showed post in comparison to pre the physical exercise intervention, enhanced TS and QF MTU capacities and some positive changes on specific mechanical markers for medial compartment knee OA. We argue that the older adults reorganised their motor task execution post exercise intervention, and made greater use of the ankle plantarflexors of their trailing leg due to the higher force-velocity potential of the TS during pushoff, thereby decreasing the need for ipsilateral hip abduction moment output. The outcome of this shift in the distribution of moment output is a more laterally directed GRF vector at the leading leg, decreasing the magnitude of external knee adduction moment during incline walking. These experimental findings corroborate with walking simulation predictions [9], [11] that a functional improvement of the TS in subjects suffering from lower-limb muscle weakness will act to reduce medial compartment mechanical loading during locomotion. Therefore, this work emphasizes the need to consider the influence of the interchange between ankle and hip joint kinetics on knee joint mechanical loading during human locomotion, and may justify the inclusion of the TS muscle in prevention programs aiming to positively influence specific mechanical markers for knee OA in the elderly. However, the study was unable to show that COMP is amenable to change in the elderly following a 14-week MTU exercise intervention. Additional studies are needed with perhaps longer intervention durations to determine the effect of improved TS and QF MTU function on serum COMP concentration and to examine the physiological relevance for the medial compartment cartilage of the biomechanical parameters studied in the elderly.

### Sharing of Materials and Data

The authors agree to make freely available any materials and data described in the current publication that may be reasonably requested for the purpose of academic, non-commercial research.

## Supporting Information

File S1 **File S1 includes Tables S1–S6. Table S1.** External eversion–inversion (Ankle_Frontal_) and dorsiflexion–plantar flexion (Ankle_Sagittal_) moments at the ankle joint in Nm kg^−1^ at different phases of the ground contact phase while incline walking determined before (Pre) and after (Post) the triceps surae and quadriceps femoris muscle-tendon unit exercise-intervention (means and SE). Ankle_Frontal_: inversion – positive, eversion – negative. Ankle_Sagittal_: plantarflexion – positive, dorsiflexion – negative. There was a significant (P<0.05) intervention (pre and post) x time window (examined intervals over the ground contact phase) effect on Ankle_Frontal_ and Ankle_Sagittal_. ^*^: Statistically significant differences between pre and post (P<0.05). **Table S2.** External adduction–abduction (Knee_Frontal_) and extension–flexion (Knee_Sagittal_) moments at the knee joint in Nm kg^−1^ at different phases of the ground contact phase while incline walking determined before (Pre) and after (Post) the triceps surae and quadriceps femoris muscle-tendon unit exercise-intervention (means and SE). Knee_Frontal_: adduction – positive, abduction – negative. Knee_Sagittal_: flexion – positive, extension – negative. There was a significant (P<0.05) intervention (pre and post) x time window (examined intervals over the ground contact phase) effect on Knee_Frontal_. ^*^: Statistically significant differences between pre and post (P<0.05). **Table S3.** External abduction–adduction (Hip_Frontal_) and flexion–extension (Hip_Sagittal_) moments at the hip joint in Nm kg^−1^ at different phases of the ground contact phase while incline walking determined before (Pre) and after (Post) the triceps surae and quadriceps femoris muscle-tendon unit exercise-intervention (means and SE). Hip_Frontal_: adduction – positive, abduction – negative. Hip_Sagittal_: extension – positive, flexion – negative. There was a significant (P<0.05) intervention (pre and post) x time window (examined intervals over the ground contact phase) effect on Hip_Frontal_. ^*^: Statistically significant differences between pre and post (P<0.05). **Table S4.** Magnitude of the ground reaction force vector (GRF_Magnitude_) and mediolateral component of the GRF (GRF_ML_) in N kg^−1^ at different phases of the ground contact phase while incline walking determined before (Pre) and after (Post) the triceps surae and quadriceps femoris muscle-tendon unit exercise-intervention (means and SE). GRF_ML_: lateral – positive, medial – negative. There was a significant (P<0.05) intervention (pre and post) x time window (examined intervals over the ground contact phase) effect on GRF_ML_. ^*^: Statistically significant differences between pre and post (P<0.05). **Table S5.** Lever arm of the ground reaction force acting about the knee joint at the frontal plane (Lever Arm Frontal_Knee_) in m at different phases of the ground contact phase while incline walking determined before (Pre) and after (Post) the triceps surae and quadriceps femoris muscle-tendon unit exercise-intervention (means and SE). Medial position of the line of action of the GRF relative to the knee joint centre – negative. There was a significant (P<0.05) intervention (pre and post) x time window (examined intervals over the ground contact phase) effect on Lever Arm Frontal_Knee_. ^*^: Statistically significant differences between pre and post (P<0.05). **Table S6.** Serum cartilage oligomeric matrix protein (COMP) concentration in U l^−1^ before (−0.5-h), immediately after (0-h), and 0.5- and 1.0-h after the 0.5-h incline walking exercise determined before (Pre) and after (Post) the triceps surae and quadriceps femoris muscle-tendon unit exercise-intervention. There was no significant muscle-tendon unit exercise intervention effect and no intervention x time effect on serum COMP concentration (P>0.05). ^1^: Statistically significant differences to −0.5-h (P<0.05). ^2^: Statistically significant differences to 0-h (P<0.05). ^3^: Statistically significant differences to 0.5-h (P<0.05).
(DOCX)
