# Supplementary material for: Effect of Exercise-Induced Enhancement of the Leg-Extensor Muscle-Tendon Unit Capacities on Ambulatory Mechanics and Knee Osteoarthritis Markers in the Elderly
Source: PLoS One. 2014 Jun 6;9(6):e99330. doi: 10.1371/journal.pone.0099330 (PMC4048280; doi:10.1371/journal.pone.0099330)
Supplement: File S1 — includes Tables S1–S6. Table S1. External eversion–inversion (AnkleFrontal) and dorsiflexion–plantar flexion (AnkleSagittal) moments at the ankle joint in Nm kg−1 at different phases of the ground contact phase while incline walking determined before (Pre) and after (Post) the triceps surae and quadriceps femoris muscle-tendon unit exercise-intervention (means and SE). AnkleFrontal: inversion – positive, eversion – negative. AnkleSagittal: plantarflexion – positive, dorsiflexion – negative. There was a significant (P<0.05) intervention (pre and post) x time window (examined intervals over the ground contact phase) effect on AnkleFrontal and AnkleSagittal. *: Statistically significant differences between pre and post (P<0.05). Table S2. External adduction–abduction (KneeFrontal) and extension–flexion (KneeSagittal) moments at the knee joint in Nm kg−1 at different phases of the ground contact phase while incline walking determined before (Pre) and after (Post) the triceps surae and quadriceps femoris muscle-tendon unit exercise-intervention (means and SE). KneeFrontal: adduction – positive, abduction – negative. KneeSagittal: flexion – positive, extension – negative. There was a significant (P<0.05) intervention (pre and post) x time window (examined intervals over the ground contact phase) effect on KneeFrontal. *: Statistically significant differences between pre and post (P<0.05). Table S3. External abduction–adduction (HipFrontal) and flexion–extension (HipSagittal) moments at the hip joint in Nm kg−1 at different phases of the ground contact phase while incline walking determined before (Pre) and after (Post) the triceps surae and quadriceps femoris muscle-tendon unit exercise-intervention (means and SE). HipFrontal: adduction – positive, abduction – negative. HipSagittal: extension – positive, flexion – negative. There was a significant (P<0.05) intervention (pre and post) x time window (examined intervals over the ground contact phase) effect on HipFro [file pone.0099330.s001.docx]

**Supplement Data:**

**Table S1:**

|  | **Ankle_Frontal_** | |  | **Ankle_Sagittal_** | |
| --- | --- | --- | --- | --- | --- |
| Interval ground contact | **Pre** | **Post** |  | **Pre** | **Post** |
| 0 to 10% | -0.005 ± 0.004 | -0.005 ± 0.004 |  | -0.037 ± 0.020 | 0.030 ± 0.017 |
| 10 to 20% | 0.012 ± 0.008 | 0.012 ± 0.009 |  | -0.169 ± 0.054 | -0.155 ± 0.040 |
| 20 to 30% | 0.045 ± 0.009 | 0.039 ± 0.010 |  | -0.368 ± 0.083 | -0.348 ± 0.067 |
| 30 to 40% | 0.057 ± 0.009 | 0.043 ± 0.009^*^ |  | -0.583 ± 0.088 | -0.574 ± 0.087 |
| 40 to 50% | 0.063 ± 0.011 | 0.041 ± 0.008^*^ |  | -0.794 ± 0.066 | -0.784 ± 0.076 |
| 50 to 60% | 0.079 ± 0.012 | 0.056 ± 0.009^*^ |  | -1.001 ± 0.042 | -0.996 ± 0.055 |
| 60 to 70% | 0.104 ± 0.013 | 0.083 ± 0.009^*^ |  | -1.182 ± 0.036 | -1.201 ± 0.040 |
| 70 to 80% | 0.130 ± 0.015 | 0.110 ± 0.011^*^ |  | -1.228 ± 0.058 | -1.311 ± 0.043^*^ |
| 80 to 90% | 0.105 ± 0.011 | 0.093 ± 0.012 |  | -0.881 ± 0.055 | -0.966 ± 0.045^*^ |
| 90 to 100% | 0.014 ± 0.003 | 0.011 ± 0.005 |  | -0.203 ± 0.021 | -0.265 ± 0.016^*^ |

**Table S2:**

|  | **Knee_Frontal_** | |  | **Knee_Sagittal_** | |
| --- | --- | --- | --- | --- | --- |
| Interval ground contact | **Pre** | **Post** |  | **Pre** | **Post** |
| 0 to 10% | 0.027 ± 0.013 | 0.000 ± 0.007^*^ |  | 0.068 ± 0.018 | 0.027 ± 0.027 |
| 10 to 20% | 0.226 ± 0.024 | 0.151 ± 0.018^*^ |  | 0.535 ± 0.037 | 0.489 ± 0.077 |
| 20 to 30% | 0.433 ± 0.030 | 0.342 ± 0.030^*^ |  | 0.713 ± 0.069 | 0.654 ± 0.109 |
| 30 to 40% | 0.468 ± 0.028 | 0.389 ± 0.038^*^ |  | 0.530 ± 0.089 | 0.427 ± 0.106 |
| 40 to 50% | 0.369 ± 0.030 | 0.300 ± 0.042^*^ |  | 0.249 ± 0.099 | 0.093 ± 0.078 |
| 50 to 60% | 0.274 ± 0.026 | 0.229 ± 0.038^*^ |  | 0.032 ± 0.088 | -0.103 ± 0.053 |
| 60 to 70% | 0.218 ± 0.026 | 0.195 ± 0.030 |  | -0.100 ± 0.075 | -0.197 ± 0.045 |
| 70 to 80% | 0.153 ± 0.027 | 0.130 ± 0.028 |  | -0.088 ± 0.059 | -0.173 ± 0.038 |
| 80 to 90% | 0.050 ± 0.019 | 0.032 ± 0.023 |  | 0.047 ± 0.024 | -0.010 ± 0.025 |
| 90 to 100% | -0.038 ± 0.003 | -0.045 ± 0.007 |  | 0.080 ± 0.009 | 0.065 ± 0.013 |

**Table S3:**

|  | **Hip_Frontal_** | |  | **Hip_Sagittal_** | |
| --- | --- | --- | --- | --- | --- |
| Interval ground contact | **Pre** | **Post** |  | **Pre** | **Post** |
| 0 to 10% | 0.084 ± 0.025 | 0.030 ± 0.022 |  | -0.602 ± 0.038 | -0.567 ± 0.068 |
| 10 to 20% | 0.505 ± 0.038 | 0.382 ± 0.027^*^ |  | -0.853 ± 0.065 | -0.976 ± 0.073 |
| 20 to 30% | 0.862 ± 0.046 | 0.735 ± 0.041^*^ |  | -0.944 ± 0.067 | -1.070 ± 0.088 |
| 30 to 40% | 0.993 ± 0.044 | 0.858 ± 0.040^*^ |  | -0.778 ± 0.072 | -0.893 ± 0.092 |
| 40 to 50% | 0.921 ± 0.041 | 0.787 ± 0.043^*^ |  | -0.540 ± 0.061 | -0.635 ± 0.090 |
| 50 to 60% | 0.813 ± 0.039 | 0.700 ± 0.043^*^ |  | -0.305 ± 0.057 | -0.405 ± 0.084 |
| 60 to 70% | 0.722 ± 0.039 | 0.659 ± 0.031^*^ |  | -0.091 ± 0.053 | -0.181 ± 0.084 |
| 70 to 80% | 0.579 ± 0.042 | 0.548 ± 0.032 |  | 0.124 ± 0.044 | 0.056 ± 0.080 |
| 80 to 90% | 0.277 ± 0.033 | 0.271 ± 0.023 |  | 0.203 ± 0.030 | 0.180 ± 0.057 |
| 90 to 100% | -0.018 ± 0.014 | -0.021 ± 0.014 |  | 0.148 ± 0.025 | 0.140 ± 0.024 |

**Table S4:**

|  | **GRF_Magnitude_** | |  | **GRF_ML_** | |
| --- | --- | --- | --- | --- | --- |
| Interval ground contact | **Pre** | **Post** |  | **Pre** | **Post** |
| 0 to 10% | 3.22 ± 0.15 | 2.82 ± 0.17 |  | -0.06 ± 0.03 | -0.01 ± 0.02 |
| 10 to 20% | 7.79 ± 0.18 | 7.67 ± 0.25 |  | -0.39 ± 0.04 | -0.28 ± 0.03^*^ |
| 20 to 30% | 10.20 ± 0.20 | 10.21 ± 0.25 |  | -0.83 ± 0.04 | -0.69 ± 0.04^*^ |
| 30 to 40% | 10.06 ± 0.26 | 9.86 ± 0.18 |  | -1.00 ± 0.04 | -0.88 ± 0.03^*^ |
| 40 to 50% | 9.06 ± 0.34 | 8.52 ± 0.24 |  | -0.90 ± 0.05 | -0.76 ± 0.04^*^ |
| 50 to 60% | 8.87 ± 0.32 | 8.41 ± 0.32 |  | -0.75 ± 0.04 | -0.63 ± 0.04^*^ |
| 60 to 70% | 9.39 ± 0.23 | 9.26 ± 0.27 |  | -0.64 ± 0.04 | -0.57 ± 0.05^*^ |
| 70 to 80% | 9.41 ± 0.36 | 9.60 ± 0.27 |  | -0.47 ± 0.03 | -0.41 ± 0.05 |
| 80 to 90% | 6.86 ± 0.34 | 7.44 ± 0.29 |  | -0.19 ± 0.03 | -0.15 ± 0.03 |
| 90 to 100% | 1.81 ± 0.11 | 1.98 ± 0.09 |  | 0.04 ± 0.01 | 0.05 ± 0.01 |

**Table S5:**

|  | **Lever Arm Frontal_Knee_** | |
| --- | --- | --- |
| Interval ground contact | **Pre** | **Post** |
| 10 to 20% | -0.026 ± 0.003 | -0.018 ± 0.002^*^ |
| 20 to 30% | -0.042 ± 0.003 | -0.033 ± 0.003^*^ |
| 30 to 40% | -0.047 ± 0.003 | -0.041 ± 0.004^*^ |
| 40 to 50% | -0.042 ± 0.003 | -0.036 ± 0.004^*^ |
| 50 to 60% | -0.032 ± 0.003 | -0.028 ± 0.004^*^ |
| 60 to 70% | -0.024 ± 0.003 | -0.023 ± 0.003 |
| 70 to 80% | -0.017 ± 0.003 | -0.015 ± 0.003 |
| 80 to 90% | -0.006 ± 0.003 | -0.005 ± 0.003 |

**Table S6:**

|  | **Serum COMP concentration** | |
| --- | --- | --- |
|  | **Pre** | **Post** |
| -0.5-h | 12.1 ± 1.2 | 11.7 ± 0.8 |
| 0-h ^1^ | 13.6 ± 1.2 | 13.2 ± 1.1 |
| 0.5-h ^2^ | 12.1 ± 1.1 | 11.4 ± 1.1 |
| 1.0-h ^1,2,3^ | 11.6 ± 1.2 | 11.1 ± 0.9 |
